# Reply: New faecal tests for colorectal cancer screening: is tumour pyruvate kinase M2 one of the options?

**DOI:** 10.1038/sj.bjc.6604040

**Published:** 2007-10-23

**Authors:** U Haug, H Brenner

**Affiliations:** 1 German Cancer Research Center (DKFZ), Division of Clinical Epidemiology and Aging Research, Bergheimer Str 20, 69115 Heidelberg, Germany

**Sir**,

We fully agree that early detection of colorectal cancer (CRC) should not only focus on cancerous lesions but also on clinically relevant precursors to CRC, bearing the potential to reduce both incidence and mortality of the disease. Nevertheless, when evaluating and characterising a screening test, it appears reasonable to clearly distinguish between sensitivity for CRC and sensitivity for precursors to CRC, as these test characteristics imply different clinical priorities. Colorectal cancer already present has high priority to be detected immediately since further growth significantly reduces chances of survival. By contrast, precursors to CRC offer the possibility to be detected by repeated testing before they become malignant at all (eg, within annual screening schemes) due to their typically long latency period. Thus, sensitivities for both lesions should be valued differently. In our study, it was only possible to estimate sensitivity of the tumour M2-PK test for CRC (which was precisely referred as that). Shastri and Stein tried to calculate a summary estimate for both lesions, which they referred to as sensitivity for colorectal neoplasia (CRN). Apart from our principal concerns regarding the meaningfulness of such a summary estimate for reasons mentioned above, it is important to do the calculations methodologically correctly, that is by considering the relative frequencies of CRC and its precursors in the general population. Shastri and Stein, however, used the estimated frequencies of CRC and its precursors in our total study population, which, given the underlying case–control design, reflects a completely arbitrary CRC/precursor ratio.

We extensively discussed the issue that controls in our study did not undergo colonoscopy and provided detailed model calculations to estimate the resulting error regarding specificity. With a prevalence of 1% of undiagnosed CRC among controls, specificity in terms of detecting CRC (ie, healthy means not having CRC) would have been underestimated by no more than 0.5% units (73.5% rather than 73.0%).

Given the comparatively low specificity of the tumour M2-PK test observed in our study, we deliberately discussed with caution whether its implementation in a population-based screening setting is justifiable, from both an ethical and an economic point of view. We miss comparable due caution in the reasoning of Shastri and Stein regarding immunological faecal occult blood testing (IFOBT), which appears to have higher specificity, but lower sensitivity compared with the tumour M2-PK test. The question which of both tests, if any, would be more appropriate for screening is very complex (see, for example, Haug and Brenner, 2005) and far from being answered by the selected data cited by Shastri and Stein. By the way, using results from the largest screening study that has been done on IFOBT (where sensitivities for advanced neoplasia and for invasive CRC were 27.1 and 65.8%, respectively) (Morikawa *et al*, 2005) would yield an estimate of sensitivity for CRN far below the level of 48% referred to as highly unacceptable by Shastri and Stein elsewhere in their letter.
